# Supplementary material for: NHR-8 and P-glycoproteins uncouple xenobiotic resistance from longevity in chemosensory C. elegans mutants
Source: eLife. 2021 Aug 27;10:e53174. doi: 10.7554/eLife.53174 (PMC8460253; doi:10.7554/eLife.53174)
Supplement: Supplementary file 2. [file elife-53174-supp2.docx]

**Supplementary File 2: List of qPCR primers used in this study**
